# Parental migration and children’s dietary diversity at home: Evidence from rural China

**DOI:** 10.1371/journal.pone.0291041

**Published:** 2023-12-07

**Authors:** Yanying Yu, Chengfang Liu, Kevin Chen, Shaoping Li

**Affiliations:** 1 China Academy for Rural Development, Zhejiang University, Hangzhou, China; 2 China Center for Agricultural Policy, School of Advanced Agricultural Sciences, Peking University, Beijing, China; 3 International Food Policy Research Institute, East and Central Asia Office, Beijing, China; Lamont-Doherty Earth Observatory, UNITED STATES

## Abstract

There is a growing literature documenting the link between parental migration and children’s health. However, few studies have explained the underlying mechanism of this observed relationship. This paper examines the effect of parental migration on children’s health through dietary diversity, using survey data collected in a less developed prefecture in South Central China in 2018. To overcome the potential endogeneity of parental migration, we instrument parental migration with the proportion of households with migrated labor force at the village level, and find that parental migration reduces children’s dietary diversity at home. Moreover, we provide suggestive evidence that the reduction in dietary diversity may attribute to significant negative separation effects whereas minimal positive income effects in migrant-sending households. This study highlights the negative effects of labor migration on the next generation’s nutrition. In those developing countries with a high prevalence of labor migration, policies that facilitate access to dietary diversity of those left-behind children are warranted.

## Introduction

A parallel phenomenon of the large rural-to-urban labor migration in China is the growing number of children who were left behind in rural villages. Since the reform and opening-up in 1978, China has witnessed a large-scale rural-urban labor migration flow. By 2021, the number of rural migrant workers has exceeded 171 million and is still expected to grow in the future years [1]. However, due to the household registration system (hukou) in China, rural migrants have limited access to public services including children’s education and other social security programs [2]. Many rural parents have to leave their children in their source villages when they move to cities for jobs. As a result, nearly 7 million rural children in 2018 were left behind with a non-parent caregiver [3].

Although a large and growing body of literature has examined the effects of parental migration on children’s health, the evidence is mixed. Some authors found that parental migration hurt children’s health because of parental absenteeism [4–7]. In contrast, other studies found that thanks to the remittance from migrated parents, parental migration played a positive role in children’s health [8–11]. Still others found no or little effect of parental migration on children’s health outcomes [12–14].

To explain the mixed evidence, we find it necessary to explore the mechanisms underlying the observed linkages or lack of linkages between parental migration and children’s health outcomes [15]. As some scholars have noted, dietary diversity might be one of these underlying mechanisms as positive associations between children’s dietary diversity and their health status have been well documented in the literature [16–19].

A close examination of the literature reveals that although some studies have examined the impact of labor migration on the dietary diversity of left-behind family members, little research has been done to examine the impact of parental migration on the dietary diversity of left-behind preschoolers in less developed rural areas of ethnic minority in China. For example, some scholars examined the effects of labor migration on the dietary diversity of left-behind family members on a whole, but they were not able to provide any evidence on the impact of migration on individual family members due to lack of individual diet records [20,21]. Several studies did examine the impact of parental migration on the dietary diversity of left-behind children [22–24], but their study contexts were either the country as a whole or the urban or township areas only. Finally, the literature paid little attention to the impact of parental migration on the dietary diversity of preschoolers, which is a critical period of children’s life course and sensitive to diet quality [25–29].

The goal of this paper is to improve our understanding about the effects of parental migration on children’s dietary diversity at home. To do so, we drew on data from a field survey covering 1,334 preschoolers from the rural areas of a less developed, ethnic minority prefecture in the south-central part of China. To overcome the potential endogeneity of parental out-migration decisions, we employed an instrumental variable approach where parental migration was instrumented with the proportion of households with migrated labor force at the village level. We found that parental migration had a significantly negative effect on children’s dietary diversity at home.

The remainder of the paper is structured as follows. The Data and variables section describes the sampling and data. The Empirical model section introduces our identification strategy along with the empirical specifications. The Empirical results section displays empirical results. We conclude in the Conclusions section.

## Data and variables

### Data

This study draws on data collected by the authors themselves from the baseline survey of a preschool nutrition pilot program conducted in the Xiangxi Autonomous Prefecture, located in the south-central region of China. With support from the World Food Programme (WFP), the survey took place in September 2018 in two then nationally designated poverty counties where rural per capita disposable income in 2017 was close to the national level in 2012 [30]. The survey covered 26 kindergartens from 15 villages, of which 10 preschools were in Longshan County and 16 were in the other Yongshun County. We surveyed the caregivers of all the children attending any one of these preschools at the survey time and obtained 1,334 questionnaires with completed answers. In addition, the team interviewed 28 preschool principals, 142 teachers, and 26 kitchen managers. The survey team collected rich individual-, household- and preschool-level information (Table 1).

**Table 1:** Descriptive statistics of key variables.

Variables	Descriptions	Mean	SD	Min	Max
**Outcome variable**					
DDS	9-group dietary diversity score of the child according to FAO; [0–9]	4.38	1.47	1.00	9.00
HDDS	12-group household dietary diversity score of the child according to FAO; [0–12]	6.61	1.69	1.00	12.00
**Key explanatory variable**					
At least one parent migrated	At least one parent migrated out for at least six months in the past year (1 = yes, 0 = no)	0.72	-	0.00	1.00
Both parents migrated	Both parents migrated out for at least six months in the past year (1 = yes, 0 = no)	0.52	-	0.00	1.00
Only one parent migrated	Only one parent migrated out for at least six months in the past year (1 = yes, 0 = no)	0.21	-	0.00	1.00
**Instrumental variable**					
Proportion of migrant-sending households	Proportion of sample households with at least one migrated member at the village level	0.80	0.11	0.37	1.00
**Child characteristics**					
Age	(month)	54.84	11.76	22.70	80.37
Girl	(1 = yes, 0 = no)	0.48	-	0.00	1.00
Non-Han ethnic minority	(1 = yes, 0 = no)	0.89	-	0.00	1.00
Picky eater	(1 = yes, 0 = no)	0.46	-	0.00	1.00
**Household characteristics**					
Father has at least a junior high school diploma	Father has at least a junior high school diploma (1 = yes, 0 = no)	0.55	-	0.00	1.00
Mother has at least a junior high school diploma	Mother has at least a junior high school diploma (1 = yes, 0 = no)	0.58	-	0.00	1.00
Pieces of durable assets	Pieces of durable assets in the child’s household	5.85	2.73	0.00	13.00
Siblings	Number of siblings of the child	0.90	0.77	0.00	5.00
The presence of at least one grandparent	Whether the child is living with at least one grandparent (1 = yes, 0 = no)	0.79	-	0.00	1.00
Household income	Annual household income per capita (log)	5.51	3.80	0.00	11.33

### The measurement of dietary diversity

Dietary diversity (DD) has been widely used in the literature as a proxy of dietary quality [31–33]. It is fairly straightforward, simply counting the number of foods or food groups consumed over a certain period [34–36]. The more food groups consumed implies higher dietary quality.

To measure children’s dietary diversity, we followed the literature and calculated each child’s Dietary Diversity Score (DDS) by counting the number of food groups that she/he consumed in the 24 hours preceding the survey date [37–40]. To do so, foods were divided into nine groups following the Guidelines for Measuring Household and Individual Dietary Diversity developed by FAO [34]. Nine food groups are starchy staples; dark green leafy vegetables; vitamin A rich fruits and vegetables; other fruits and vegetables; organ meat; meat and fish; eggs; legumes, nuts and seeds; and milk and milk products. We collected dietary information by asking primary caregivers to answer questions like “Did the child eat any staple foods such as rice soup, porridge, noodles, steamed bun, or rice over the past 24 hours at home?”. However, unlike previous studies measurng children’s dietary diversity both at home and at school, we focused on home diet’s DDS to avoid the possibility that school lunches would average out the measures of dietary diversity for all.

There is a small proportion of preschoolers (2.3%) who participated in the survey on the survey date but missed information on home-diet as they did not have meals at home the day before the survey date. When we compared children with missing diet records and those with complete information, our data showed that there is little systematic difference between these two groups of students in terms of most child and household characteristics (S1 Table). The results showed that the sample is mostly balanced. However, we did find that children with missing home-diet information are less likely to have both parents migrated and live with at least one grandparent. In our analyses, we imputed missing diet information with the sample average.

### Parental migration status

The key explanatory variable of interest in this study is the status of parental migration. Following National Bureau of Statistics, we identified a parent as a migrant if he/she had worked out of his/her township for at least 6 months over the last year. With this information, we screened three types of parental migration. The first one is called at least one parent migrated, which is indicated by a dummy variable that takes the value of one when at least one parent migrated last year and zero otherwise. The second one is called both parents migrated, which is indicated by another dummy variable that takes the value of one when both parents migrated last year and zero otherwise. The last one is called only one parent migrated, which is indicated by still another dummy variable that takes the value of one when only the mother or the father migrated last year and zero otherwise. In this way, we seek to examine whether different combinations of parental migration have different impacts on children’s dietary diversity at home. Our data show 45.6% of sample preschoolers live in households where both parents migrated out, higher than the average of 35.5% in rural areas of Hunan [41].

### Covariates

In addition, we also constructed control variables to measure variables at the child, household, preschool levels that might affect children’s dietary diversity. Following the literature, we took into account four variables at the child level, including the child’s age, gender, whether she/he is a Non-Han ethnic minority, and whether she/he is a picky eater [20,42–45]. We controlled for six household characteristics, including whether the child’s father and mother has completed at least junior high school education, the number of the child’s siblings, pieces of durable assets, the presence of at least one grandparent, and household income per capita [20,43,45–47]. We also controlled preschool dummies for two reasons. One was to account for the substitution effects led by school feeding, as the quality of home diets might be compromised by preschool lunches. The other was to take into account all community-specific characteristics that could influence the possibility of out-migration and the access to food variety.

## Empirical model

To estimate the effects of parental migration on children’s dietary diversity at home, we specified the following empirical model:

$${DDS}_{i}=\alpha_{1}+\beta_{1}{PM}_{i}+\delta_{1}X_{i}+\varepsilon_{i}$$ (1)

where DDS_*i*_ denotes the dietary diversity score of the child *i*; *PM*_*i*_ is a dummy variable that takes the value of one if at least one of the two parents migrated and zero otherwise. *β*_1_ is the coefficient of interest. ***X***_***i***_ is a vector of child-, household-level control variables, and preschool dummies, which we described above. *ε*_*i*_ is an error term. We clustered standard errors at the class level.

OLS estimate of *β*_1_ from Model (1) would be biased due to the potential endogeneity of parental migration from at least two sources. One is reverse causality as parents might take their children’s nutritional status into account when making their out-migration decisions. Instinctively, earning more money to feed kids better may be one of the motivations for parents to migrate. The other source is omitted variable bias as Model (1) might have missed some unobservable factors that simultaneously influence the migration decision of parents and the dietary diversity of children. For example, parents who care more about children’s nutritional status are more likely to migrate to earn more money for the children and also devote more time and efforts to improve children’s dietary diversity. In that case, OLS estimates might overestimate the positive effect of parental migration. Another example is when the family suffers from unobservable shocks, the children may be offered a poor diet whereas parents migrate to enhance the family’s resistance against shocks, which leads to exaggerated negative effects of parental migration on children’s dietary diversity by the OLS estimates.

To overcome the endogeneity of parental migration, we take an instrumental variable (IV) approach. Following the literature [48,49], we instrument parental migration status with the social network of migrants from the same community, which is highly associated with personal migration decisions but has no direct impact on children’s dietary diversity unless through parental migration. Specifically, for each child i in village j, we calculate the proportion of sample migrant-sending households in village j excluding household i, and use it as an instrumental variable of parental migration. Our justifications for the validity of this IV is that the proportion of sample migrant-sending households in the village influences parents’ out-migration decisions, whereas it does not directly link to children’s dietary diversity, given that a vector of child-, household-level control variables, and preschool dummies have been taken into consideration. We then apply the IV approach and implement the following two-stage least squared estimation. The first stage goes as follows:

$${PM}_{i}=\alpha_{2}+\gamma {IV}_{i}+\theta X_{i}+\varepsilon_{i}$$ (2)

With the predicted value of parental migration from stage one ($\hat{PM}$), we specify the second stage as follows:

$${DDS}_{i}=\alpha_{3}+\beta_{2}{\hat{PM}}_{i}+\delta_{2}X_{i}+\varepsilon_{i}$$ (3)

## Empirical results

Table 2 compared statistics of children by their parents’ migration status. Our data showed that compared with children living with both parents, children with at least one parent migrated had a lower dietary diversity at home. Specifically, the average DDS for children exposing to parental migration ranged from 4.20 among children with both parents migrated to 4.51 among those with only one parent migrated, against 4.61 among those living with both parents. The average DDS of children with at least one migrated parent were statistically lower than those living with both parents (P<0.01). When we considered the specific combinations of parental migration, the differences of dietary diversity scores were observed between children with both parents migrated and those living with both parents (P<0.01). However, children with only one parent migrated did not show significant disadvantages in terms of dietary diversity scores. It seemed that parental migration might have reduced children’s dietary diversity, especially when both parents migrated out.

**Table 2:** Comparison of groups with migrated parent and non-migrated parent.

	At least one parent migrated	Both parents migrated	Only one parent migrated	No parent migrated
	M (*SD*)	M (*SD*)	M (*SD*)	M (*SD*)
	(1)	(2)	(3)	(4)
DDS	4.29*** (1.44)	4.20*** (1.43)	4.51 (1.46)	4.61 (1.51)
**Child characteristics**				
Age	55.59*** (11.71)	56.23*** (11.60)	54.00 (11.85)	52.87 (11.66)
Girl	0.49 (0.50)	0.48 (0.50)	0.50 (0.50)	0.48 (0.50)
Non-Han ethnic minority	0.89 (0.32)	0.89 (0.32)	0.89 (0.32)	0.90 (0.31)
Picky eater	0.45 (0.50)	0.45 (0.50)	0.47 (0.50)	0.48 (0.50)
**Household characteristics**				
Father has at least a junior high school diploma		0.51*** (0.50)	0.50*** (0.50)	0.55** (0.50)	0.65 (0.48)
Mother has at least a junior high school diploma	0.53*** (0.50)	0.50*** (0.50)	0.59*** (0.49)	0.70 (0.46)
Pieces of durable assets	5.34*** (2.59)	5.15*** (2.53)	5.81*** (2.67)	7.21 (2.65)
Siblings	0.88 (0.78)	0.84** (0.74)	0.97 (0.88)	0.95 (0.74)
The presence of at least one grandparent	0.80 (0.40)	0.85*** (0.35)	0.68** (0.47)	0.77 (0.42)
Household income	5.26*** (3.76)	5.25*** (3.72)	5.28*** (3.86)	6.18 (3.80)
Observations	967	689	278	367

*Notes*: Means in Column (1)-(3) are separately compared with that in Column (4) using two-tailed t-tests. *, **, and ***, 10, 5, and 1% statistical significances, respectively.

We also assessed the similarities and differences in other characteristics between children of migrants and children of non-migrants in Table 2. For the most part, many individual-level characteristics of these two groups were not statistically different from each other, with one exception. For examples, compared with their peers with both parents around, children with any migrated parents, regardless of their parental migration combinations, shared similar gender and ethnicity composition. However, children with any migrated parents, especially when both parents migrated, tended to be elder than children of non-migrants. In the meantime, our data also showed that children with different parental migration status appeared to be different from each other in some household characteristics. In particular, compared with their peers with both parents around, children with any migrated parents tended to come from households with lower socioeconomic status in terms of durable assets and household income per capita, and their parents tended to be less educated. To control for these observable differences between children with and without migrated parents, we included them in Models (1)-(3) as control variables.

Table 3 presented OLS and IV estimates of the impact of parental migration on children’s dietary diversity scores at home. We started with the OLS estimations of Eq (1), assuming parental migration as an exogenous independent variable. The OLS model yielded a negative but insignificant coefficient on the migration variable. However, the OLS estimates were likely to be biased because they did not account for the endogeneity of migration.

**Table 3:** OLS and IV estimation results on the impact of parental migration on children’s Dietary Diversity Scores (DDS).

	Model 1: OLS	Model 2: IV
Variable	DDS	DDS
At least one parent migrated	-0.13(0.09)	-0.54***(0.20)
**Child characteristics**		
Age	-0.00(0.00)	-0.00(0.00)
Girl	0.11(0.08)	0.11(0.08)
Non-Han ethnic minority	0.00(0.14)	-0.02(0.14)
Picky eater	-0.17**(0.08)	-0.18**(0.08)
**Household characteristics**		
Father has at least a junior high school diploma	0.29***(0.09)	0.29***(0.09)
Mother has at least a junior high school diploma	0.21**(0.08)	0.19**(0.09)
Pieces of durable assets	0.07***(0.02)	0.05***(0.02)
Siblings	-0.04(0.06)	-0.06(0.06)
The presence of at least one grandparent	0.00(0.11)	0.02(0.11)
Household income	0.02*(0.01)	0.02*(0.01)
Preschool dummy	YES	YES
Constant	3.58***(0.41)	3.99***(0.48)
Observations	1,334	1,334
R-squared	0.12	0.11
F Statistics for Weak Identification		22.13

*Notes*: Robust standard errors clustered at the class level are in parentheses. *, **, and ***, 10, 5, and 1% statistical significances, respectively.

To address the potential endogeneity of parental migration, we then estimated Eqs (2) and (3) and presented the results in Model 2 of Table 3. The F-value for the first-stage equation (22.13) was larger than the rule of thumb value of 10, indicating that the instrument is not weak [50]. In the second stage, unlike the findings from OLS estimations, we found significantly negative effects of parental migration on children’s dietary diversity scores at home. Specifically, children with at least one parent migrated scored 0.54 points lower in DDS than their peers with both parents around, and the coefficient was statistically significant at one percent level (Model 2, Table 3).

To explore the possibility that different combinations of parental migration might exert different impacts on children’s DDS, we replaced the at least one parent migrated dummy variable with two dummy variables (namely, only one parent migrated and both parents migrated) and reran Eqs (1) to (3) in Table 4. The results showed that parental migration, especially with both parents involved did undermine children’s DDS after accounting for the endogeneity of migration. Specifically, compared with children living with both parents, children with only one migrated parent and children with both parents migrated scored 0.47 (P<0.1) and 0.63 points (P<0.01) lower in DDS, respectively. The first-stage F-values in both cases, were higher than the rule of thumb value of 10 (25.92 for Model 2 and 16.58 for Model 4), excluding the possibility of weak instruments. Although in the OLS model, labor migration with only one parent involved yielded positive effects on children’s DDS, the estimate was statistically insignificant (Model 1).

**Table 4:** OLS and IV estimation results for different combinations of parental migration.

	Model 1: OLS	Model 2: IV	Model 3: OLS	Model 4: IV
Variable	DDS	DDS	DDS	DDS
Only one parent migrated	0.06(0.12)	-0.47*(0.27)		
Both parents migrated			-0.23**(0.10)	-0.63***(0.18)
**Child characteristics**				
Age	0.00(0.00)	0.00(0.00)	0.00(0.00)	0.00(0.00)
Girl	0.07(0.11)	0.08(0.11)	0.18**(0.09)	0.18**(0.09)
Non-Han ethnic minority	-0.24(0.23)	-0.27(0.23)	0.15(0.14)	0.13(0.14)
Picky eater	-0.21*(0.12)	-0.20*(0.11)	-0.23**(0.10)	-0.23**(0.10)
**Household characteristics**				
Father has at least a junior high school diploma	0.20(0.14)	0.21(0.14)	0.31***(0.11)	0.30***(0.11)
Mother has at least a junior high school diploma	0.31**(0.14)	0.30**(0.14)	0.13(0.10)	0.10(0.10)
Pieces of durable assets	0.08***(0.03)	0.06**(0.03)	0.07***(0.02)	0.05**(0.02)
Siblings	-0.10(0.08)	-0.11(0.07)	-0.05(0.08)	-0.08(0.08)
The presence of at least one grandparent	0.12(0.16)	0.07(0.16)	0.09(0.12)	0.15(0.13)
Household income	0.02(0.01)	0.02(0.01)	0.03*(0.01)	0.02(0.01)
Preschool dummy	YES	YES	YES	YES
Constant	3.12***(0.56)	3.53***(0.63)	3.34***(0.45)	3.70***(0.48)
Observations	645	645	1,056	1,056
R-squared	0.19	0.17	0.13	0.12
F Statistics for Weak Identification		25.92		16.58

*Notes*: Robust standard errors clustered at the class level are in parentheses. *, **, and ***, 10, 5, and 1% statistical significances, respectively.

As a robustness check, we also used an alternative measure of dietary quality and reran the empirical specification. Specifically, we used the 12-group Household Dietary Diversity Score (HDDS) developed by FAO (2013) [34]. Compared with the 9-group DDS, the 12-group HDDS include three more groups, namely oils /fats, sweets and spices/condiments/beverages. Since these three additional food groups contained in the 12-group HDDS do not contribute to the essential micronutrient density of the diet, the 12-group HDDS is not better than the 9-group DDS when assessing the nutritional quality of individuals’ diet [34]. And the IV regression results from using the 12-group HDDS as the dependent variable remain substantially the same as those from using the 9-group DDS (S2 Table), suggesting our results were robust to alternative measure of dietary quality.

But why there were negative effects of parental migration on children’s dietary diversity at home? One explanation was that the adverse effect of lower parental care was relatively large compared to the income effect exerted by remittances. If labor migration created income-growth linkages [51], the migrant-sending households that experienced eased budget constraints might increase nutritional investment on children [52]. However, parental absence tended to undermine children’s nutrition if there was no perfect substitution for parental care [53,54]. Therefore, to explain the mechanism underlying parental migration and children’s dietary diversity, we specially tested whether or not parental migration would change children’s food expenditures and primary caregiver’s nutrition knowledge which respectively embodied income effects and absence effects exerted by parental migration in Table 5.

**Table 5:** IV results for food expenditures and caregiver’s nutrition knowledge.

Variable	Model 1	Model 2	Model 3	Model 4	Model 5
	Total food expenditures	Snacks	Fruits	Meat, eggs and milk	Nutrition knowledge score
	Coef (SE)	Coef (SE)	Coef (SE)	Coef (SE)	Coef (SE)
At least one parent migrated^a^	-2.33	4.39	-11.14	1.18	-5.98***
	(37.88)	(10.02)	(15.08)	(17.98)	(1.63)
One parent migrated^b^	87.60	15.82	13.58	35.01	-5.22**
	(73.27)	(15.28)	(24.84)	(33.88)	(2.43)
Both parents migrated^c^	-33.84	1.44	-20.92*	-13.10	-6.55***
	(34.39)	(11.19)	(12.29)	(16.02)	(1.62)

*Note*: Robust standard errors clustered at the class level are in parentheses; The models are conditional on child characteristics, household characteristics and preschool dummies. *, **, and ***, 10, 5, and 1% statistical significances, respectively; Food expenditures are winsorised at the 2.5th percentile and 97.5th percentile to set extreme outliers equal to less extreme values.

^a^ *N* = 1,334

^b^ *N* = 645

^c^ *N* = 1,056.

We first replaced the dependent variables in Eq (3) with four variables measuring children’s monthly food expenditures: total expenditure on food, expenditure on snacks, expenditure on fruits and expenditure on meat, eggs and milk. We did not find any positive effects of parental migration on children’s food expenditures (Models 1 to 4). In 11 out of 12 cases, the coefficients were not statistically different from zero. Interestingly, when both parents out migrated, children’s expenditure on fruits decreased by 20.92 yuan per month compared to that of children living with both parents. And it was significant at the 10% level. The results generally suggested that income effects exerted by labor migration did not always associate with improved investment on the nutrition of the children left behind.

We next replaced the dependent variables in Eq (3) with nutrition knowledge scores of the children’s primary caregiver. A test developed in collaboration with experts from the Institute of Food and Nutrition Development, Ministry of Agriculture and Rural Affairs was used to measure primary caregivers’ nutrition knowledge scores (with a full score of 100). The results showed that compared to children of non-migrants, children of migrants were more likely to be taken care of by a primary caregiver with less nutrition knowledge. Specifically, compared to the primary caregiver of children living with both parents, the primary caregiver of children with any parent migrated, children with only one parent migrated and children with both parents migrated scored 5.98 (P<0.01), 5.22 (P<0.05) and 6.55 (P<0.01) points lower in terms of nutrition knowledge scores, respectively.

## Conclusions

Parental migration is expected to exert both positive and negative effects on the dietary diversity of children who are left behind. The net effects of parental migration on children’s dietary diversity is largely an empirical question. In this paper, we explored the net effects of parental migration on children’s dietary diversity at home, based on a cross-sectional dataset collected in two then nationally designated poverty counties in Xiangxi, province of Hunan, China. To overcome the potential endogeneity of parental migration as parental migration may depend on unobservable household characteristics that also affect children’s dietary diversity, we instrumented parental migration with the proportion of households with migrated labor force at the village level.

We found a significantly negative effect of parental migration on children’s dietary diversity at home. Moreover, compared to parental migration with only one parent involved, parental migration with both parents involved yielded more salient negative effects on children’s dietary diversity at home. These findings may help explain the negative effects of parental migration on children’s health outcomes found in previous studies.

As a suggestive explanation of the negative effects of parental migration on children’s dietary diversity at home, we found that the adverse effect of parental absence was relatively large compared to the income effect exerted by remittances. When exposing to parental migration, whether with one parent involved or with both parents involved, the children were tended to be nurtured by a caregiver with lower nutrition knowledge acquisition. In addition, although labor migration plays an important role in boosting household income in the rural areas of China, our results indicated that it did not increase food expenditures on children left-behind in our sample.

As dietary diversity is critical to children’s nutrition and health, our research findings that established a negative relationship between parental migration and children’s dietary diversity bear at least two policy implications. First, interventions and programs targeted at improving left-behind children’s living environments and alleviating the negative effects brought by parental absence need to be a priority. For example, school feeding program might help to narrow the nutrition gap between children lack of parental guardianship and children with better parental care. Second, policy attention is deserved to encourage the rural left-behind children to reunite with their parents in cities. To this end, measures are warrant for reducing the institutional restrictions on the mobility of these children.

We acknowledge at least two limitations of our study. First, constrained by our data, we are only able to measure children’s dietary diversity with DDS based on a 24-h recall, which cannot capture dietary variations across seasons. Although such a 24-h DDS could help to minimize recall error, DDS based on longer valid recall periods would help further examine children’s dietary habit. Second, our sample are from the rural areas of two less developed counties with high concentration of ethnic minority, cautions need to be taken when trying to generalize our research findings to other contexts.

## Supporting information

S1 Table Comparison of groups with missing information and complete information. (DOCX)

S2 Table IV estimation results for household dietary diversity scores (HDDS). (DOCX)

S1 File (DOCX)

## References

[1] National Bureau of Statistics of China. The 2021 Statistical Communique on National Economic and Social Development. 2022 Feb 28 [cited 20 July 2022]. Available from: http://www.gov.cn/xinwen/2022-02/28/content_5676015.htm (in Chinese).

[2] Song Y. What should economists know about the current Chinese hukou system? China Economic Review. 2014;29:200–12.

[3] Ministry of Civil Affairs of China. Chart: Data on left-behind children in rural areas in 2018. 2018 Sep 1 [cited 20 July 2022]. Available from: http://www.mca.gov.cn/article/gk/tjtb/201809/20180900010882.shtml (in Chinese).

[4] de Brauw A, Mu R. Migration and the overweight and underweight status of children in rural China. Food Policy. 2011;36(1):88–100.

[5] Gao Y, Li LP, Kim JH, Congdon N, Lau J, Griffiths S. The impact of parental migration on health status and health behaviours among left behind adolescent school children in China. BMC Public Health. 2010;10(1):56. doi: 10.1186/1471-2458-10-56 20128901 PMC2829003

[6] Lei L, Liu F, Hill E. Labour Migration and Health of Left-Behind Children in China. The Journal of Development Studies. 2018;54(1):93–110.

[7] Meng X, Yamauchi C. Children of Migrants: The Cumulative Impact of Parental Migration on Children’s Education and Health Outcomes in China. Demography. 2017;54(5):1677–1714. doi: 10.1007/s13524-017-0613-z 28936632

[8] Azzarri C, Zezza A. International migration and nutritional outcomes in Tajikistan. Food Policy. 2011;36(1):54–70.

[9] Carletto C, Covarrubias K, Maluccio JA. Migration and child growth in rural Guatemala. Food Policy. 2011;36(1):16–27.

[10] de Brauw A. Migration and child development during the food price crisis in El Salvador. Food Policy. 2011;36(1):28–40.

[11] Mu R, de Brauw A. Migration and young child nutrition: evidence from rural China. Journal of Population Economics. 2015;28(3):631–657.

[12] Guo Q, Sun W, Wang Y. Effect of Parental Migration on Children’s Health in Rural China. Review of Development Economics. 2017;21(4):1132–1157.

[13] Xu H, Xie Y. The Causal Effects of Rural-to-Urban Migration on Children’s Well-being in China. European Sociological Review. 2015;31(4):502–519. 10.1093/esr/jcv009 PMC4507435 26207080

[14] Zhou C, Sylvia S, Zhang L, Luo R, Yi H, Liu C, et al. China’s left-behind children: Impact of parental migration on health, nutrition, and educational outcomes. Health Affairs. 2015;34(11):1964–1971. doi: 10.1377/hlthaff.2015.0150 26526256

[15] Antman FM. International Migration and Gender Discrimination among Children Left Behind. American Economic Review. 2011;101(3):645–649. doi: 10.1257/aer.101.3.645 23239896 PMC3518870

[16] Aboagye RG, Seidu A, Ahinkorah BO, Arthur-Holmes F, Cadri A, Dadzie LK, et al. Dietary diversity and undernutrition in children aged 6–23 months in sub-saharan africa. Nutrients. 2021;13(10):3431. doi: 10.3390/nu13103431 34684435 PMC8537414

[17] Arimond M, Ruel MT. Dietary diversity is associated with child nutritional status: Evidence from 11 demographic and health surveys. Journal of Nutrition. 2004;134(10):2579–2585. doi: 10.1093/jn/134.10.2579 15465751

[18] Rah JH, Akhter N, Semba RD, Pee SD, Bloem MW, Campbell AA, et al. Low dietary diversity is a predictor of child stunting in rural Bangladesh. European Journal of Clinical Nutrition. 2010;64(12):1393–1398. doi: 10.1038/ejcn.2010.171 20842167

[19] Thorne-Lyman AL, Valpiani N, Sun K, Semba RD, Klotz CL, Kraemer K, et al. Household dietary diversity and food expenditures are closely linked in rural Bangladesh, increasing the risk of malnutrition due to the financial crisis. Journal of Nutrition. 2010;140(1):182S–188S. doi: 10.3945/jn.109.110809 19923385

[20] Min S HOU L, Hermann W, Huang J, Mu Y. The impact of migration on the food consumption and nutrition of left-behind family members: Evidence from a minority mountainous region of southwestern China. Journal of Integrative Agriculture. 2019;18(8):1780–1792.

[21] Nguyen MC, Winters P. The impact of migration on food consumption patterns: The case of Vietnam. Food Policy. 2011;36(1):71–87.

[22] Gibson J, McKenzie D, Stillman S. What happens to diet and child health when migration splits households? Evidence from a migration lottery program. Food Policy. 2011;36(1):7–15.

[23] Ning M, Chang HH. Migration decisions of parents and the nutrition intakes of children left at home in rural China. Agricultural Economics. 2013;59(10):467–477.

[24] Zhang N, Bécares L, Chandola T. A multilevel analysis of the relationship between parental migration and left-behind children’s macronutrient intakes in rural China. Public Health Nutrition. 2016;19(11):1913–1927. doi: 10.1017/S1368980015003341 26641518 PMC4974629

[25] Heckman JJ. Skill Formation and the Economics of Investing in Disadvantaged Children. Science. 2006;312(5782):1900–1902. doi: 10.1126/science.1128898 16809525

[26] Grantham-McGregor S, Cheung YB, Cueto S, Glewwe P, Richter L, Strupp B, et al. Child development in developing countries 1—Developmental potential in the first 5 years for children in developing countries. Lancet. 2007;369(9555):60–70. 17208643 10.1016/S0140-6736(07)60032-4 PMC2270351

[27] Rosales FJ, Reznick JS, Zeisel SH. Understanding the role of nutrition in the brain and behavioral development of toddlers and preschool children: identifying and addressing methodological barriers. Nutritional Neuroscience. 2009;12(5):190–202. doi: 10.1179/147683009X423454 19761650 PMC2776771

[28] Walker SP, Wachs TD, Gardner JM, Lozoff B, Wasserman GA, Pollitt E, et al. Child development in developing countries 2—Child development: risk factors for adverse outcomes in developing countries. Lancet. 2007;369(9556):145–157. 17223478 10.1016/S0140-6736(07)60076-2

[29] Walker SP, Wachs TD, Grantham-McGregor S, Black MM, Nelson CA, Huffman SL, et al. Child Development 1: Inequality in early childhood: risk and protective factors for early child development. Lancet. 2011;378(9799):1325–1338. 21944375 10.1016/S0140-6736(11)60555-2

[30] Bi J, Liu C, Li S, He Z, Chen K, Luo R, et al. Dietary diversity among preschoolers: A cross-sectional study in poor, rural, and ethnic minority areas of central south china. Nutrients. 2019;11(3):558. doi: 10.3390/nu11030558 30845662 PMC6471221

[31] Hatløy A, Torheim LE, Oshaug A. Food variety—A good indicator of nutritional adequacy of the diet? A case study from an urban area in Mali, West Africa. European Journal of Clinical Nutrition. 1998;52(12):891–898. 9881884 10.1038/sj.ejcn.1600662

[32] Ruel MT. Operationalizing Dietary Diversity: A Review of Measurement Issues and Research Priorities. Journal of Nutrition. 2003;133(11):3911S–26S. doi: 10.1093/jn/133.11.3911S 14672290

[33] Stein AD. 90th Anniversary Commentary: Dietary Diversity Is the Cornerstone of Good Nutrition. Journal of Nutrition. 2018;148(10):1683–1685. doi: 10.1093/jn/nxy128 30281101

[34] FAO (Food and Agriculture Organization of the United Nations). Guidelines for measuring household and individual dietary diversity. 2010. Available from: https://www.fao.org/3/i1983e/i1983e.pdf.

[35] Hoddinott J, Yohannes Y. Dietary diversity as a food security indicator. 2002. Available from: https://www.fantaproject.org/sites/default/files/resources/DietaryDiversity-HH-FS-Indicator-2002.pdf.

[36] Ruel MT. Is Dietary Diversity an Indicator of Food Security or Dietary Quality? A Review of Measurement Issues and Research Needs. Food and Nutrition Bulletin. 2003;24(2):231–232. doi: 10.1177/156482650302400210 12891828

[37] Savy M, Martin-Prével Y, Sawadogo P, Kameli Y, Delpeuch F. Use of variety diversity scores for diet quality measurement: relation with nutritional status of women in a rural area in Burkina Faso. European Journal of Clinical Nutrition. 2005;59(5):703–16. doi: 10.1038/sj.ejcn.1602135 15867942

[38] Steyn N, Nel J, Nantel G, Kennedy G, Labadarios D. Food variety and dietary diversity scores in children: are they good indicators of dietary adequacy? Public Health Nutrition. 2006;9(5):644–650. doi: 10.1079/phn2005912 16923296

[39] Kennedy GL, Pedro MR, Seghieri C, Nantel G, & Brouwer I. Dietary diversity score is a useful indicator of micronutrient intake in non-breast-feeding Filipino children. Journal of Nutrition. 2007;137(2): 472–477. doi: 10.1093/jn/137.2.472 17237329

[40] Zhao W, Yu K, Tan S, Zheng Y, Zhao A, Wang P, Zhang Y. Dietary diversity scores: An indicator of micronutrient inadequacy instead of obesity for chinese children. BMC Public Health, 2017;17(1):440–11. doi: 10.1186/s12889-017-4381-x 28499361 PMC5429576

[41] Hunan Provincial Bureau of Statistics. Child development monitoring report of Hunan Province in 2018. 2019 Aug 16 [cited 2 July 2023]. Available from: http://tjj.hunan.gov.cn/hntj/tpxw/201908/t20190819_5414863.html (in Chinese).

[42] Aurino E. Do boys eat better than girls in India? Longitudinal evidence on dietary diversity and food consumption disparities among children and adolescents. Economics and Human Biology. 2017;25:99–111. doi: 10.1016/j.ehb.2016.10.007 27810442

[43] Cabalda AB, Rayco-Solon P, Solon JAA, Solon FS. Home gardening is associated with filipino preschool children’s dietary diversity. Journal of the American Dietetic Association. 2011;111(5):711–715. doi: 10.1016/j.jada.2011.02.005 21515117

[44] Carruth BR, Skinner J, Houck K, Moran J III, Coletta F, Ott D. The phenomenon of “picky eater”: A behavioral marker in eating patterns of toddlers. Journal of the American College of Nutrition. 1998;17(2):180–186. doi: 10.1080/07315724.1998.10718744 9550462

[45] Gelli A, Margolies A, Santacroce M, Roschnik N, Twalibu A, Katundu M, et al. Using a Community-Based Early Childhood Development Center as a Platform to Promote Production and Consumption Diversity Increases Children’s Dietary Intake and Reduces Stunting in Malawi: A Cluster-Randomized Trial. Journal of Nutrition. 2018;148(10):1587–1597. doi: 10.1093/jn/nxy148 30204916 PMC6168702

[46] Appannah G, Murray K, Trapp G, Dymock M, Oddy WH, Ambrosini GL. Dietary pattern trajectories across adolescence and early adulthood and their associations with childhood and parental factors. American Journal of Clinical Nutrition. 2021;113(1):36–46. doi: 10.1093/ajcn/nqaa281 33181820

[47] Tang Z. What makes a difference to children’s health in rural china? parental migration, remittances, and social support. Chinese Sociological Review. 2016;49(2):1–21.

[48] Chang H, Dong X, MacPhail F. Labor Migration and Time Use Patterns of the Left-behind Children and Elderly in Rural China. World Development. 2011;39(12):2199–2210.

[49] Meyerhoefer CD, Chen CJ. The effect of parental labor migration on children’s educational progress in rural china. Review of Economics of the Household. 2011;9(3):379–396.

[50] Staiger D, Stock JH. Instrumental Variables Regression with Weak Instruments. Econometrica. 1997;65(3):557–586.

[51] Taylor EJ. The New Economics of Labour Migration and the Role of Remittances in the Migration Process. International Migration. 1999;37(1):63–88. doi: 10.1111/1468-2435.00066 12322077

[52] Yang D. Migrant Remittances. The Journal of Economic Perspectives. 2011;25(3):129–151.

[53] Zhang N, Bécares L, Chandola T, Callery P. Intergenerational differences in beliefs about healthy eating among carers of left-behind children in rural China: A qualitative study. Appetite. 2015;95:484–491. doi: 10.1016/j.appet.2015.08.024 26299714

[54] Liu B, Qing P, Xiao S, Liao F. The Influence of Grandparents’ Indulgence on the Health Status of the Left-behind Children In Rural Areas from the Perspective of Food Consumption: A Case Study from Hubei Province. Chinese Rural Economy. 2019;1:32–46. (in Chinese)

