# Supplementary material for: Parental migration and children’s dietary diversity at home: Evidence from rural China
Source: PLoS One. 2023 Dec 7;18(12):e0291041. doi: 10.1371/journal.pone.0291041 (PMC10703207; doi:10.1371/journal.pone.0291041)
Supplement: S1 Table — (DOCX) [file pone.0291041.s001.docx]

**S1 Table. Comparison of groups with missing information and complete information**

|  | Children with missing home-diet information | Children with complete information | P-value of difference test  H0: (1) = (2) |
| --- | --- | --- | --- |
|  | M (SD) | M (SD) |  |
|  | (1) | (2) |  |
| **Parental migration status** |  |  |  |
| At least one parent migrated | 0.61 (0.50) | 0.73 (0.45) | 0.16 |
| Both parents migrated | 0.32 (0.48) | 0.52 (0.50) | 0.03 |
| Only one parent migrated | 0.29 (0.46) | 0.21 (0.40) | 0.26 |
| **Child characteristics** |  |  |  |
| Age | 54.48 (12.48) | 54.85 (11.74) | 0.86 |
| Girl | 0.55 (0.51) | 0.48 (0.50) | 0.46 |
| Non-Han ethnic minority | 0.94 (0.25) | 0.89 (0.32) | 0.41 |
| Picky eater | 0.42 (0.50) | 0.46 (0.50) | 0.63 |
| **Household characteristics** |  |  |  |
| Father has at least a junior high school diploma | 0.58 (0.50) | 0.55 (0.50) | 0.72 |
| Mother has at least a junior high school diploma | 0.55 (0.51) | 0.58 (0.49) | 0.74 |
| Pieces of durable assets | 5.94 (2.72) | 5.85 (2.74) | 0.87 |
| Siblings | 0.81 (0.75) | 0.90 (0.77) | 0.50 |
| The presence of at least one grandparent | 0.65 (0.49) | 0.80 (0.40) | 0.04 |
| Household income | 5.68 (3.84) | 5.51 (3.80) | 0.81 |
| Observations | 31 | 1,303 | 1,334 |
