# Supplementary material for: Parental migration and children’s dietary diversity at home: Evidence from rural China
Source: PLoS One. 2023 Dec 7;18(12):e0291041. doi: 10.1371/journal.pone.0291041 (PMC10703207; doi:10.1371/journal.pone.0291041)
Supplement: S2 Table — (DOCX) [file pone.0291041.s002.docx]

**S2 Table.** **IV estimation results for household dietary diversity scores (HDDS)**

|  | Model 1 | Model 2 | Model 3 |
| --- | --- | --- | --- |
| Variable | HDDS | HDDS | HDDS |
| At least one parent migrated | -0.71^***^(0.21) |  |  |
| One parent migrated |  | -0.54^*^(0.33) |  |
| Both parents migrated |  |  | -0.86^***^(0.20) |
| **Child characteristics** |  |  |  |
| Age | 0.00(0.00) | 0.01^*^(0.00) | 0.00(0.00) |
| Girl | 0.11(0.09) | 0.09(0.12) | 0.19^*^(0.10) |
| Non-Han ethnic minority | -0.08(0.16) | -0.37(0.25) | 0.04(0.17) |
| Picky eater | -0.16^*^(0.08) | -0.16(0.13) | -0.23^**^(0.10) |
| **Household characteristics** |  |  |  |
| Father has at least a junior high school diploma | 0.32^***^(0.10) | 0.23(0.15) | 0.33^***^(0.13) |
| Mother has at least a junior high school diploma | 0.18^*^(0.10) | 0.32^**^(0.15) | 0.06(0.12) |
| Pieces of durable assets | 0.06^***^(0.02) | 0.09^***^(0.03) | 0.05^**^(0.03) |
| Siblings | -0.04(0.07) | -0.08(0.08) | -0.10(0.09) |
| The presence of at least one grandparent | -0.01(0.13) | 0.13(0.19) | 0.08(0.15) |
| Household income | 0.02(0.01) | 0.02(0.02) | 0.02(0.02) |
| Preschool dummy | YES | YES | YES |
| Constant | 6.15^***^(0.52) | 5.39^***^(0.69) | 6.09^***^(0.54) |
| Observations | 1,334 | 645 | 1,056 |
| R-squared | 0.12 | 0.19 | 0.12 |
| F Statistics for Weak Identification | 22.13 | 25.92 | 16.58 |

*Notes:* Robust standard errors clustered at the class level are in parentheses. ^*^, ^**^, and ^***^, 10, 5, and 1% statistical significances, respectively.
