# Supplementary material for: Parental migration and children’s dietary diversity at home: Evidence from rural China
Source: PLoS One. 2023 Dec 7;18(12):e0291041. doi: 10.1371/journal.pone.0291041 (PMC10703207; doi:10.1371/journal.pone.0291041)
Supplement: S1 File — (DOCX) [file pone.0291041.s003.docx]

**S1 Table. Comparison of groups with missing information and complete information**

|  | Children with missing home-diet information | Children with complete information | P-value of difference test  H0: (1) = (2) |
| --- | --- | --- | --- |
|  | M (SD) | M (SD) |  |
|  | (1) | (2) |  |
| **Parental migration status** |  |  |  |
| At least one parent migrated | 0.61 (0.50) | 0.73 (0.45) | 0.16 |
| Both parents migrated | 0.32 (0.48) | 0.52 (0.50) | 0.03 |
| Only one parent migrated | 0.29 (0.46) | 0.21 (0.40) | 0.26 |
| **Child characteristics** |  |  |  |
| Age | 54.48 (12.48) | 54.85 (11.74) | 0.86 |
| Girl | 0.55 (0.51) | 0.48 (0.50) | 0.46 |
| Non-Han ethnic minority | 0.94 (0.25) | 0.89 (0.32) | 0.41 |
| Picky eater | 0.42 (0.50) | 0.46 (0.50) | 0.63 |
| **Household characteristics** |  |  |  |
| Father has at least a junior high school diploma | 0.58 (0.50) | 0.55 (0.50) | 0.72 |
| Mother has at least a junior high school diploma | 0.55 (0.51) | 0.58 (0.49) | 0.74 |
| Pieces of durable assets | 5.94 (2.72) | 5.85 (2.74) | 0.87 |
| Siblings | 0.81 (0.75) | 0.90 (0.77) | 0.50 |
| The presence of at least one grandparent | 0.65 (0.49) | 0.80 (0.40) | 0.04 |
| Household income | 5.68 (3.84) | 5.51 (3.80) | 0.81 |
| Observations | 31 | 1,303 | 1,334 |

**S2 Table.** **IV estimation results for household dietary diversity scores (HDDS)**

|  | Model 1 | Model 2 | Model 3 |
| --- | --- | --- | --- |
| Variable | HDDS | HDDS | HDDS |
| At least one parent migrated | -0.71^***^(0.21) |  |  |
| One parent migrated |  | -0.54^*^(0.33) |  |
| Both parents migrated |  |  | -0.86^***^(0.20) |
| **Child characteristics** |  |  |  |
| Age | 0.00(0.00) | 0.01^*^(0.00) | 0.00(0.00) |
| Girl | 0.11(0.09) | 0.09(0.12) | 0.19^*^(0.10) |
| Non-Han ethnic minority | -0.08(0.16) | -0.37(0.25) | 0.04(0.17) |
| Picky eater | -0.16^*^(0.08) | -0.16(0.13) | -0.23^**^(0.10) |
| **Household characteristics** |  |  |  |
| Father has at least a junior high school diploma | 0.32^***^(0.10) | 0.23(0.15) | 0.33^***^(0.13) |
| Mother has at least a junior high school diploma | 0.18^*^(0.10) | 0.32^**^(0.15) | 0.06(0.12) |
| Pieces of durable assets | 0.06^***^(0.02) | 0.09^***^(0.03) | 0.05^**^(0.03) |
| Siblings | -0.04(0.07) | -0.08(0.08) | -0.10(0.09) |
| The presence of at least one grandparent | -0.01(0.13) | 0.13(0.19) | 0.08(0.15) |
| Household income | 0.02(0.01) | 0.02(0.02) | 0.02(0.02) |
| Preschool dummy | YES | YES | YES |
| Constant | 6.15^***^(0.52) | 5.39^***^(0.69) | 6.09^***^(0.54) |
| Observations | 1,334 | 645 | 1,056 |
| R-squared | 0.12 | 0.19 | 0.12 |
| F Statistics for Weak Identification | 22.13 | 25.92 | 16.58 |

*Notes:* Robust standard errors clustered at the class level are in parentheses. ^*^, ^**^, and ^***^, 10, 5, and 1% statistical significances, respectively.
